# Early optimization in finger dexterity of skilled pianists: implication of transcranial stimulation

**DOI:** 10.1186/1471-2202-14-35

**Published:** 2013-03-16

**Authors:** Shinichi Furuya, Michael A Nitsche, Walter Paulus, Eckart Altenmüller

**Affiliations:** 1 Institute for Music Physiology and Musicians’ Medicine, Hannover University of Music, Drama and Media, Hannover, Germany; 2 Department of Clinical Neurophysiology, Georg-August-University Göttingen, Göttingen, Germany

**Keywords:** tDCS, Fine motor control, Neuroplasticity, Motor learning, Skilled finger movements

## Abstract

**Background:**

It has been shown that non-invasive transcranial direct current stimulation (tDCS) facilitates motor functions in healthy adults and stroke patients. However, little is known about neuroplastic changes induced by tDCS in highly-trained individuals. Here we addressed this issue by assessing the effect of tDCS on dexterity of finger movements in healthy adult pianists. Twelve pianists practiced bimanual keystrokes in an in-phase manner while bilateral tDCS (left anodal/right cathodal or vice versa) of the primary motor cortex was performed. Before and after stimulation, each participant was asked to perform the trained successive keystrokes, and to repetitively strike a key with each of the fingers as fast and accurate as possible while keeping the remaining fingers immobilized voluntarily.

**Results:**

In contrast to previous findings in untrained individuals, tDCS yielded overall no apparent improvement of fine control of finger movements in the professional pianists. However, for some movement features, pianists who commenced training at later age demonstrated larger improvements of fine motor control following tDCS.

**Conclusions:**

These findings, in combination with lack of any correlation between the age at which pianists commenced the training and motor improvements for sham stimulation conditions, supports the idea that selectively late-started players benefit from tDCS, which we interpret as early optimization of neuroplasticity of the motor system.

## Background

Plasticity of the nervous system is a mechanism that enables us to acquire novel motor and perceptual skills. Non-invasive transcranial direct current stimulation (tDCS) provides a clue to investigate neuroplasticity of the sensory-motor system. It yields NMDA receptor-dependent cortical plasticity through subthreshold polarization of neuronal membranes [1,2]. Anodal tDCS over the motor cortex facilitates excitation of neurons [3-5] and enhances hand motor functions [6-9]. Yet, these findings were limited to young and elderly individuals with no history of extensive motor training [10,11] and stroke patients [12,13]. It is therefore not known whether tDCS similarly elicits boosting effects on motor functions of highly-skilled individuals such as athletes and musicians.

Musicians have been investigated as an ideal model to probe neuroplasticity for several reasons: they commence musical training at an early age, practice intensely over many years while acquiring increasingly complex skills and furthermore perform these skills with a high degree of arousal, motivation and positive emotions [14-16]. A previous study demonstrated a larger improvement of spatial tactile acuity after perceptual training in musicians than in non-musicians [17]. This was interpreted as metaplasticity, i.e. as an improved capacity for plastic reorganization in skilled musicians. Understanding metaplasticity of musicians is important for unraveling pathophysiological mechanism of focal dystonia, a neurological disorder eliciting maladaptive neuroplastic changes that degrade the motor system involved in fine motor control. In keeping with this, a case report showed an improvement of fine motor control in a pianist suffering from focal dystonia with tDCS over days [18], which was interpreted as an induction of beneficial brain plasticity using tDCS. It is therefore likely to predict that plastic changes induced by stimulating the motor system of musicians might be possibly even large for musicians who commenced musical training early.

However, tDCS elicits stronger impacts on motor function of the non-dominant hand compared with the dominant one in healthy humans [8,9], which implicates use-dependent optimization of neuroplasticity of the motor system. An alternative hypothesis therefore proposes a ceiling effect at neuroplasticity of musicians due to years of intensive motor training from childhood. This predicts a lack of substantial improvements accomplished by tDCS of the motor cortex of musicians, and/or even smaller enhancing effects on individuals who commenced musical training earlier.

The present study aimed to address this issue by assessing effects of bi-hemispheric tDCS on fine control of finger movements at the right and left hands of expert pianists, a key feature of dexterous use of the hand [6,19]. Previous studies that investigated untrained individuals showed that bi-hemispheric tDCS could elicit a stimulation polarity-dependent increase and decrease in the excitability of both hemispheres [20,21], and thereby facilitate fine control of finger movements, sometimes to a larger extent than uni-hemispheric tDCS [22]. Evidence for lack of enhancement of fine control of finger movements following tDCS, and in particular of a smaller gain for players who began musical training earlier, would argue against the notion that early musical training provides superior neuroplasticity of the motor system, whereas prominent effects of tDCS would argue for enhanced plasticity.

## Methods

### Participants

Twelve expert pianists (4 males, age: 24–29 yrs old, age of commencing piano education: 3–8 yrs old, all right handed: Edinburgh’s test score ranges from 40 to 80% [23]) participated in the experiments. All participants had a professional piano education at universities, and had won prizes at international and/or national piano competitions. They practiced the piano for 3.75 ± 1.14 hours per day. The experiment was carried out according to the Declaration of Helsinki and all participants signed informed consents before the experiment. The study was approved by the local ethical committee located in Hannover Medical University.

### Experimental design

The participants were asked to participate in three experiments with different stimulation protocols including right anodal/left cathodal, right cathodal/left anodal, or sham tDCS (“RaLc”, “RcLa”, or “sham”). The rationale for using bi-hemispheric motor cortex tDCS instead of uni-hemispheric stimulation was that stronger effects were reported for bihemispheric stimulation [22], possibly due to suppression of inter-hemispheric inhibition to the cortex stimulated by anodal tDCS because of the excitability-diminishing effects of contralateral cathodal tDCS. To minimize the current shunt between the electrodes over the scalp, the location of the electrodes was carefully selected so that the distance of the edges of the electrodes was at least 6 cm [24]. Each experiment for each participant was separated by more than two weeks from each other in order to minimize any carry-over or retention effect of the stimulation. The order of the stimulation protocols was balanced across participants, and also double-blinded so that the experimenter and participant could not know the ongoing stimulation protocol.

The present experiment consisted of three successive sessions; pretest, training, and posttest. During the training session, two active electrodes were put on C3 and C4 locations (= primary motor cortexes), which were identified using the international 10–20 electroencephalogram system. The tDCS was applied throughout the session (24 min). For the “RaLc” and “sham” conditions, the cathodal and anodal electrode was placed on the left and right motor cortex, respectively, and vice versa for the “RcLa” condition. The intensity of stimulation was 2 mA for the “RaLc” and “RcLa” protocols, and 0.2 mA for the “sham” protocol. tDCS was induced through water-soaked sponge electrodes (surface 35 cm^2^) and delivered by a battery-driven constant-current stimulator (eldith GmbH, Germany). This method of tDCS has already been used in numerous studies and was shown to be safe [10]. Three participants reported mild skin irritations initially, which disappeared within a few minutes.

During the training session, participants were asked to successively strike four adjacent keys (right: G-F-E-D, left: C-D-E-F) with the index, middle, ring and little fingers bimanually in an in-phase manner in synchrony with a metronome (3 strokes per beat, 100 beats per minute, inter-keystroke interval = 200 ms). The whole training session consisted of 8 sub-sessions, each of which consisted of the bimanual playing for 150 sec and subsequent rest for 30 sec (3 min × 8 sessions). A sequence was played with *legato* touch, meaning that a key is not released until the next key is depressed. Each participant was asked to play at the loudness of 70 MIDI velocity (roughly corresponding to *mezzo-forte*). The pilot study confirmed no occurrence of muscular fatigue throughout the training session.

During the pretest and posttest sessions, the independence of individuated finger movements was evaluated by the fastest constrained finger tapping task. For each of the right and left hands, each participant was asked to repetitively strike a piano key with one of the four fingers as fast and accurate as possible for 6 seconds while keeping the adjacent keys depressed with the remaining digits so as to immobilize them. The finger tapping was performed in a random order across fingers, and repeated twice for each finger of each hand. In addition, each participant played the trained sequence of keystrokes for 8 seconds with each of the right and left hands in synchrony with a metronome (inter-keystroke interval = 200 ms) in order to evaluate training-induced changes in temporal accuracy of sequential finger movements.

### Data acquisition and analysis

We recorded musical instrument digital interface (MIDI) data from the keyboard by using a custom-made script in LabVIEW (National Instruments), running at 500 Hz [25]. From MIDI data, we derived information on the time the key was depressed and released. The mean and standard deviation of the inter-keystroke interval and finger-key contact duration across strokes were computed for each trial based on MIDI data derived during the fastest tapping test for each of the four fingers at each of the right and left hands. Each of the variables evaluated was averaged across the two trials. Here the mean of the inter-keystroke interval and finger-key contact duration represents speed of repetitive finger movements and quickness of lifting a finger, respectively, and standard deviation of these variables represent their variability.

### Statistics

For the variables derived from each of the tasks of performing the trained sequence of keystrokes and fastest tapping with each of the fingers, a three-way analysis of variance (ANOVA) with repeated measures with the factors “training” (pretest/ posttest), “stimulation protocol” (RaLc/ RcLa/ sham), and “hand” (right/ left) was carried out. Tukey post-hoc tests were performed in case of significant results of the ANOVA. Furthermore, in order to probe meta-plasticity of the motor cortex, a correlation analysis was performed between each of the variables and age when the player started to play the piano. Statistical analyses were carried out using R statistical software (Ver. 2.15.2) for the ANOVAs and the statistical toolbox of MATLAB (Mathworks co.) for the correlation analysis and post-hoc analyses including bootstrap and robust regression (see Results).

## Results

### Results of ANOVA

Table 1 and 2 summarizes group mean and results of the three-way repeated measures ANOVA for the variables evaluated in both tasks (i.e. trained sequence of keystrokes and fastest finger tapping). Overall, no training effects were evident. Although some variables showed a significant main effect and/or interaction effect of training, Tukey post-hoc tests did not identify any differences between pre- and posttest. Moreover, the results indicate no apparent impact of tDCS on temporal accuracy of the trained sequential finger movements as well as both speed and accuracy of the fastest finger tapping movements. These findings suggest robustness of the motor system responsible for skilled finger movements against any short-term training with tDCS.

**Table 1:** The group mean and standard deviation of the variables evaluated across pianists

				**Right hand**						**Left hand**					
				**RaLc**		**RcLa**		**sham**		**RaLc**		**RcLa**		**sham**	
				**Pre**	**Post**	**Pre**	**Post**	**Pre**	**Post**	**Pre**	**Post**	**Pre**	**Post**	**Pre**	**Post**
Trained keystrokes	IKI SD		mean	11.0	9.8	11.7	10.3	11.5	10.1	9.9	10.2	11.2	10.3	10.8	9.7
			SD	3.7	2.4	3.8	2.7	3.2	2.5	1.8	2.2	1.8	2.5	2.8	3.0
	CD SD		mean	17.1	16.2	19.0	16.4	16.4	16.7	14.2	14.7	15.9	14.0	15.0	17.5
			SD	3.9	4.2	4.6	3.2	3.9	4.8	3.7	3.9	3.6	3.6	3.6	9.9
Finger tapping	IK-mean	I	mean	144.2	142.9	146.2	143.0	140.9	144.2	154.5	154.0	153.1	153.5	153.3	155.6
			SD	10.6	13.9	13.2	10.9	11.8	13.4	13.5	14.6	14.0	11.4	12.6	14.2
		M	mean	156.1	153.4	152.7	153.2	153.3	154.6	164.3	167.0	167.0	166.3	165.0	168.2
			SD	14.3	17.5	17.2	14.7	11.5	26.0	12.8	14.9	12.9	14.7	10.6	13.9
		R	mean	184.5	179.7	175.6	174.4	178.1	172.4	189.1	187.0	199.3	188.1	187.2	181.4
			SD	37.4	25.2	26.6	23.7	14.3	8.9	27.7	26.5	61.5	27.9	25.5	26.0
		L	mean	158.6	161.8	160.1	165.6	155.5	158.6	175.4	168.8	172.6	168.6	170.2	169.2
			SD	11.7	13.8	13.4	18.1	10.7	13.4	16.7	14.3	13.2	12.1	10.5	10.9
	IKI-SD	I	mean	12.7	11.3	13.1	11.9	13.0	12.4	15.8	13.0	18.1	14.6	17.7	15.1
			SD	6.2	4.2	5.8	4.3	6.2	5.5	5.2	6.1	9.3	5.4	8.5	8.2
		M	mean	24.2	21.2	25.9	22.8	21.5	22.3	18.6	19.0	24.0	21.5	19.4	18.2
			SD	14.4	11.5	13.6	12.7	15.9	17.5	6.9	6.6	10.0	9.8	8.1	5.9
		R	mean	34.7	33.3	30.9	32.9	31.9	31.7	34.0	28.2	33.5	28.5	26.0	25.5
			SD	21.2	24.3	24.2	22.2	16.6	20.3	19.8	17.4	29.5	19.9	20.7	17.0
		L	mean	19.9	25.8	21.3	24.4	21.2	25.0	17.9	17.0	18.5	23.7	18.8	20.7
			SD	8.3	14.8	9.1	18.1	10.0	17.9	7.0	7.6	6.5	8.4	7.8	8.2
	CD-mean	I	mean	42.8	43.7	45.9	41.9	41.8	44.4	56.9	51.9	58.9	57.9	50.7	57.5
			SD	12.0	15.1	14.2	9.2	12.8	11.1	17.4	15.0	15.4	18.6	12.5	16.3
		M	mean	55.0	52.7	61.0	58.3	63.7	52.5	65.3	62.5	65.8	66.7	62.2	62.8
			SD	8.5	13.2	22.4	18.4	31.3	14.2	17.8	16.6	20.6	22.4	19.3	19.0
		R	mean	84.5	76.2	75.6	65.2	68.2	62.9	90.2	78.0	80.8	78.9	78.2	79.2
			SD	43.5	32.4	22.9	16.5	18.9	19.1	23.0	21.4	19.6	25.8	22.5	21.0
		L	mean	59.4	64.0	61.5	62.0	58.8	60.7	78.2	68.9	69.8	61.4	71.6	66.1
			SD	19.3	27.0	19.7	18.5	18.2	20.2	24.3	18.0	22.5	17.3	21.3	19.0
	CD-SD	I	mean	8.3	7.2	7.3	6.7	7.8	8.2	13.2	10.0	13.4	11.2	10.6	11.0
			SD	4.8	3.2	3.1	3.6	4.9	5.7	6.1	4.3	3.9	3.9	4.3	3.7
		M	mean	13.7	11.2	13.1	11.3	13.4	12.0	14.	15.0	16.0	14.8	14.9	12.7
			SD	6.2	6.3	5.6	5.9	9.1	10.2	5.8	6.3	5.6	8.7	6.5	4.7
		R	mean	20.0	19.2	17.9	14.2	17.7	14.5	18.5	17.9	20.7	19.3	17.6	18.7
			SD	12.6	11.7	6.4	4.6	8.7	5.3	5.6	7.2	7.8	11.6	7.2	6.5
		L	mean	12.5	15.8	14.7	12.6	10.6	11.4	15.5	14.9	14.8	14.7	15.9	15.1
			SD	5.5	12.1	5.2	5.3	4.5	4.4	5.8	5.1	5.9	6.3	5.4	6.5

IKI: inter-keystroke interval, CD: finger-key contact duration.

I, M, R, L indicates the index, middle, ring and little fingers, respectively.

Pre, post indicates pretest and posttest, respectively.

**Table 2:** Results of three-way ANOVA with repeated measures (F and P values)

				**Hand**	**Training**	**Montage**	**H × T**	**H × M**	**T × M**	**H × T × M**
				**(1, 11)**	**(1, 11)**	**(2, 22)**	**(1, 11)**	**(2, 22)**	**(2, 22)**	**(2, 22)**
Trained keystrokes	IKI SD		F	1.15	46.68	0.75	1.83	0.11	0.68	0.80
			p	0.31	**0.00**	0.48	0.20	0.90	0.52	0.46
	CD SD		F	3.63	0.16	0.75	0.93	2.10	4.01	0.10
			p	0.08	0.70	0.49	0.35	0.15	**0.03**	0.90
Finger tapping	IKI-mean	I	F	55.64	0.01	0.06	0.57	0.87	2.15	0.67
			p	**0.00**	0.91	0.94	0.47	0.43	0.14	0.52
		M	F	16.08	0.14	0.01	0.39	0.27	0.26	0.73
			p	**0.00**	0.71	0.99	0.55	0.77	0.77	0.49
		R	F	2.37	5.39	0.73	0.14	1.66	0.38	0.22
			p	0.15	**0.04**	0.49	0.71	0.21	0.69	0.80
		L	F	12.07	0.00	1.37	4.70	1.08	0.50	1.02
			p	**0.01**	0.99	0.27	0.05	0.36	0.61	0.38
	IKI-SD	I	F	3.63	7.75	1.31	5.98	0.29	0.09	0.02
			p	0.08	**0.02**	0.29	**0.03**	0.75	0.92	0.98
		M	F	1.10	1.26	1.25	0.11	0.19	0.46	0.52
			p	0.32	0.28	0.31	0.75	0.83	0.64	0.60
		R	F	0.54	1.67	1.30	4.60	2.07	1.31	0.21
			p	0.48	0.22	0.29	0.06	0.15	0.29	0.81
		L	F	2.00	2.13	0.92	0.72	1.46	0.32	1.63
			p	0.18	0.17	0.41	0.41	0.25	0.73	0.22
	CD-mean	I	F	34.00	0.00	0.80	0.03	0.47	3.13	1.72
			p	**0.00**	0.99	0.46	0.86	0.63	0.06	0.20
		M	F	1.59	3.02	1.09	6.69	0.40	0.57	2.46
			p	0.23	0.11	0.35	**0.03**	0.68	0.58	0.11
		R	F	1.89	3.99	2.61	0.95	0.69	2.16	1.39
			p	0.20	0.07	0.10	0.35	0.51	0.14	0.27
		L	F	10.52	2.11	1.12	5.74	3.07	0.16	0.53
			p	**0.01**	0.17	0.35	**0.04**	0.07	0.85	0.60
	CD-SD	I	F	19.54	4.34	0.08	2.23	2.89	1.98	0.38
			p	**0.00**	0.06	0.92	0.16	0.08	0.16	0.69
		M	F	1.12	18.40	0.10	0.53	0.60	0.20	0.81
			p	0.31	**0.00**	0.91	0.48	0.56	0.82	0.46
		R	F	0.42	3.14	0.74	3.66	2.02	1.25	1.06
			p	0.53	0.10	0.49	0.08	0.16	0.31	0.36
		L	F	2.46	0.00	0.91	1.89	2.10	0.64	1.31
			p	0.15	0.95	0.42	0.20	0.15	0.54	0.29

A number in a parenthesis indicates a degree of freedom.

A bold number indicates p < 0.05.

H: hand, T: training, M: montage.

IKI: inter-keystroke interval, CD: finger-key contract duration.

I, M, R, L indicates the index, middle, ring and little fingers, respectively.

Some variables showed a main effect of hand, and an interaction effect of hand and training (Table 2). The effects indicated faster keystrokes and shorter finger-key contact duration and a smaller variability during the finger tapping task for the right hand compared with the left hand. However, the respective post-hoc tests did not determine any hand-dependent training effects.

### Correlation analysis

**Figure 1:**
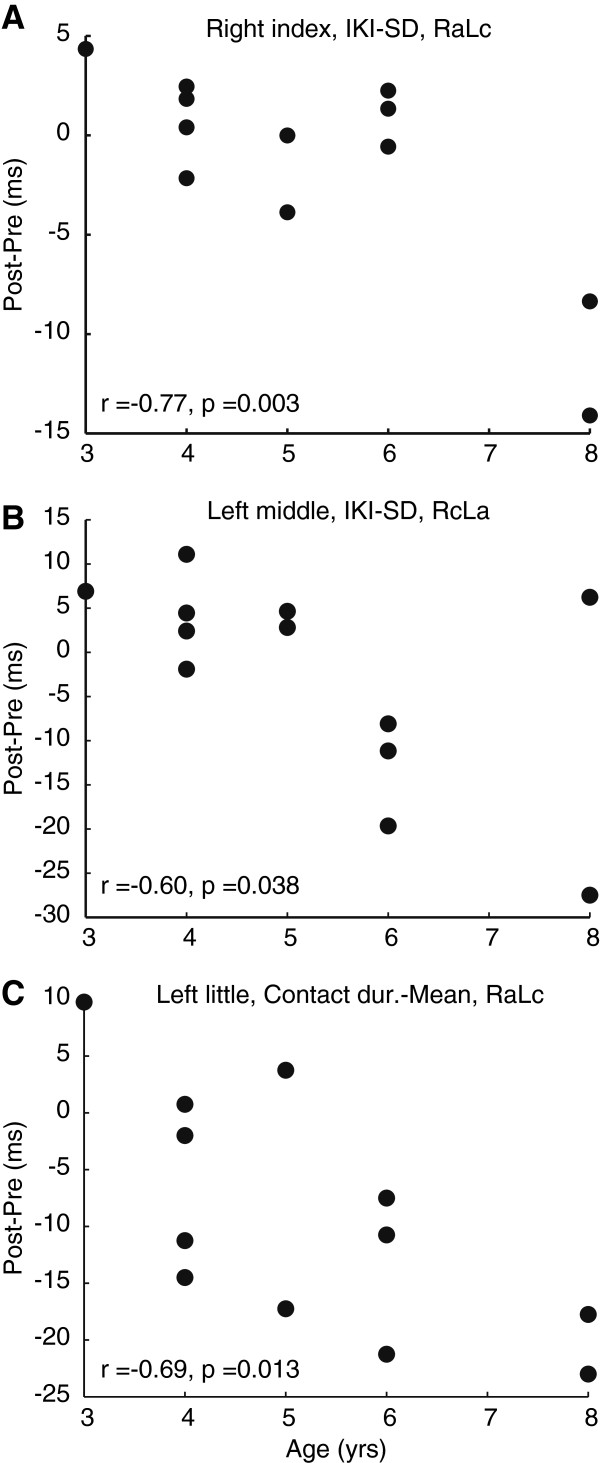
**Relation between the age at which each player started to play the piano and the difference between pre- and post-tests in terms of variability of the inter-keystroke interval at the right index finger at the RaLc montage (A), variability of the inter-keystroke interval at the left middle finger at the RcLa montage (B), and average of the finger-key contact duration at the left little finger at the RaLc montage (C), respectively.** A negative value indicates a decrease (improvement) following the stimulation. Each dot indicates an individual player.

Table 3 summarizes the correlation coefficients between the age of starting piano training and each of the variables evaluated at the four fingers of both hands. Figure 1 selectively displays the results with significant correlations, which includes the relation between the age when each player started to play the piano and a difference between pre- and post-tests in terms of variability of the inter-keystroke interval at the right index finger at the RaLc montage (A), variability of the inter-keystroke interval at the left middle finger at the RcLa montage (B), and average of the finger-key contact duration at the left little finger at the RaLc montage (C), respectively. Here, a negative value indicates a decrease of variability or finger-key contact duration following the training session. A clear negative relation was discernible for these three variables with significant correlation coefficient values. This negative relation indicates that players who started piano playing later showed stronger effect of stimulation, such as larger decreases of movement variability and shorter finger-key contact duration. Indeed, cathodal stimulation over the motor cortex yielded a decrease of the temporal variability of keystrokes at the contra-lateral hand, whereas the anodal simulation resulted in quicker release of the finger at the contra-lateral hand. None of the other stimulation protocols and variables yielded significant correlations.

**Table 3:** Correlation coefficients between the age of commencing piano training and individual movement variables during the repetitive keystrokes

		**Index**				**Middle**				**Ring**				**Little**			
		**IKI-mean**	**IKI-SD**	**CD-mean**	**CD-SD**	**IKI-mean**	**IKI-SD**	**CD-mean**	**CD-SD**	**IKI-mean**	**IKI-SD**	**CD-mean**	**CD-SD**	**IKI-mean**	**IKI-SD**	**CD-mean**	**CD-SD**
Right	RaLc	0.24	**−0.77**	0.21	−0.45	0.38	−0.05	−0.17	−0.27	0.40	−0.44	−0.25	0.22	−0.51	−0.08	−0.47	−0.19
	RcLa	−0.41	−0.50	−0.31	−0.20	0.05	0.04	0.15	−0.24	0.04	0.09	0.07	−0.29	−0.47	−0.32	−0.06	−0.06
	sham	0.39	0.42	−0.27	0.05	−0.21	0.16	0.27	0.00	0.02	−0.37	0.43	0.07	−0.32	−0.20	−0.10	0.16
Left	RaLc	−0.05	−0.04	−0.55	−0.50	0.25	0.33	0.33	0.46	−0.17	0.02	−0.37	0.35	−0.35	−0.15	**−0.69**	0.03
	RcLa	−0.31	0.01	−0.14	0.02	−0.14	**−0.60**	0.15	−0.22	−0.07	−0.35	−0.20	0.11	−0.25	−0.06	−0.08	0.41
	sham	−0.33	−0.36	0.36	−0.14	−0.20	0.40	0.30	0.43	−0.03	0.41	−0.06	0.28	0.21	0.27	−0.28	−0.03

IKI: inter-keystroke interval, CD: finger-key contact duration.

A number in bold indicates a significant correlation (p < 0.05).

In order to increase our confidence in the reliability of the results derived from the correlation analysis that used only twelve datasets, we further performed (1) a robust regression analysis [26] and (2) a bootstrap procedure [27]. The robust regression analysis evaluates the significance of covariation between two variables even in the presence of outliers, which can matter when the number of datasets is limited. The result show that the p value of the correlation between the age of starting piano playing and a performance difference between pre- and post-tests was 0.005, 0.002, and 0.020 for variability of the inter-keystroke interval at the right index finger at the RaLc montage, variability of the inter-keystroke interval at the left middle finger at the RcLa montage, and average of the finger-key contact duration at the left little finger at the RaLc montage, respectively. These results of the robust regression analysis support the notion that the age when each player started to play the piano accounted for the impact of tDCS on these three movement variables. The bootstrap procedure was also performed to identify the 95^th^ percentile confidence interval of the correlation coefficient. Both the upper and lower confidence limits of the correlation coefficient derived from 1000 bootstrap samples were −0.94 and −0.05, -0.94 and 0.02, and −0.90 and −0.35 for the three variables mentioned above. All correlation coefficient values (i.e. Figure 1) were situated between lower and upper confidence interval limits. Thus these results confirm the negative values of these correlation coefficients independent of distribution of a population.

### Gender effect

To assess a gender effect on both the current motor task and training effects, a four-way repeated measure ANOVA with unequal sample sizes was performed for each of the variables evaluated. Neither a main effect of gender nor an interaction effect between gender and training was detected for any tested variable (p > 0.05).

## Discussion

The present study attempted to identify whether pianists with intensive training in childhood could benefit from transcranial direct current stimulation (tDCS) to induce plastic changes that facilitate dexterous finger movements. Overall, no apparent improvement of individuated finger movements was evident in terms of speed and accuracy following any types of the stimulation with piano practice at both the right and left hand for the whole group under study. Similarly, a lack of any tDCS effects was found for temporal accuracy of sequential finger movements during performing the motor task used for the training. This is in contrast to previous findings of bi-hemispheric tDCS studies that demonstrated facilitation of skilled finger movements even without finger training [22]. The present finding is therefore suggestive for a ceiling effect at the motor system of professional pianists. However, the age at which pianists commenced piano training was positively correlated with the improvement of the finger movements by practice combined with stimulation, which indicates a smaller gain for pianists who started to play piano earlier. Taken together, these findings indicate robustness of the motor system of pianists against the tDCS intervention, being likely to reflect an early optimization of neuroplasticity.

Neuroplasticity of the motor system of musicians has been previously investigated by transcranial magnetic stimulation (TMS) [28]. They found that musicians with early commencement of training showed larger plasticity. However, they included different instrumentalists, and argued that this finding was attributed to differences in the age of commencement of musical training across instrumentalists. The present study, for the first time, investigated this issue by studying a homogeneous group (i.e. classical pianists), and straightforwardly identified the relation between plasticity of musician’s motor system and age at which commencing the training, which was revealed specifically by the real stimulation but not the sham one. This is in favor of the assumption of an early optimization (before age of 8) of central nervous representations of fine motor skills [29,30], which are stabilized when training through years starts in early childhood [31].

The meta-plasticity of the present pianists who started later in life to play the piano can be associated with a potential risk of focal dystonia of musicians (FD). FD is associated with abnormal functional and structural changes at the cortical and subcortical regions responsible for fine motor control [8,32]. One of the predominant triggering factors is repetition of movements, which can lead maladaptive changes at these regions [33,34]. The robustness of neuroplasticity of early-beginning pianists thus predicts their low risk of occurrence of focal dystonia. Indeed, a recent study identified that musician who commenced musical training before the age seven showed lower risk of focal dystonia [35].

A decrease in temporal variability of movements particularly of the late-started pianists was evident at the hand contra-lateral to the cathodal electrode, whereas their finger-key contact duration was shortened at the hand contra-lateral to the anodal one. During the constrained finger-tapping task, the tapping motion can be interfered by immobilizing the adjacent fingers due to the inter-digit dependence at the biomechanical and neural levels [36]. The cathodal stimulation that decreases neuronal excitation may aid in suppressing this interference from the immobilized fingers to the moving finger, being thereby likely to facilitate the temporal accuracy of the tapping motions. By contrast, the anodal stimulation that increases neuronal excitation via depolarizing the deeper layers of the motor cortex can enhance quickness of the transition of movement direction from flexion and extension or simply agility of the finger movement, which would shorten the finger-key contact duration. These findings implicate distinct mechanisms responsible for temporal and spatial control of individuated finger movements. The observation of correlation only at some particular fingers and variables is puzzling, and needs to be investigated in future studies by using neuroimaging (fMRI, PET) and transcranial magnetic stimulation (TMS).

The present study entails several limitations to be elaborated upon in future studies. First, a larger number of participants would ensure more reliable results of the correlation analysis. Second, stimulation with longer duration and over days might elicit more pronounced effects on motor performance. A recent study with stroke patients indeed showed improvements of the motor functions over days of stimulation (Lindenburg et al. 2012), which suggests a possibility of accumulative effects of tDCS. Third, the physiological effects of tDCS should be explored by assessing motor evoked potentials (MEP) evoked by TMS on the motor cortex before and after the tDCS in future studies, to rule out insufficient physiological effects in this specific subjects group.

## Conclusions

The present study demonstrated that effects of tDCS on finger dexterity of skilled pianists depended on the age at which players commenced piano playing. This finding implicates early optimization of neuroplasticity of the motor cortex responsible for skilled finger movements.

## Competing interests

The authors declare no disclosure of financial interests and potential conflict of interest.

## Authors’ contributions

SF participated in the design of the study, carried out the experiments, analyzed data, performed the statistical analysis, and drafted the manuscript. MN participated in the design and coordination of the study and helped to draft the manuscript. WP participated in the design and coordination of the study and helped to draft the manuscript. EA participated in the design and coordination of the study, drafted the manuscript, and helped to draft the manuscript. All authors read and approved the final manuscript.
